# Salt Stress Encourages Proline Accumulation by Regulating Proline Biosynthesis and Degradation in Jerusalem Artichoke Plantlets

**DOI:** 10.1371/journal.pone.0062085

**Published:** 2013-04-29

**Authors:** Zengrong Huang, Long Zhao, Dandan Chen, Mingxiang Liang, Zhaopu Liu, Hongbo Shao, Xiaohua Long

**Affiliations:** 1 Jiangsu Key Lab of Marine Biology, College of Resources and Environmental Sciences, Nanjing Agricultural University, Nanjing, China; 2 Key Laboratory of Coastal Biology and Bioesources Utilization, Yantai Institute of Coastal Zone Research (YIC), Chinese Academy of Sciences (CAS), Yantai, China; 3 Institute for Life Sciences, Qingdao University of Science and Technology, Qingdao, China; University of South Florida College of Medicine, United States of America

## Abstract

Proline accumulation is an important mechanism for osmotic regulation under salt stress. In this study, we evaluated proline accumulation profiles in roots, stems and leaves of Jerusalem artichoke (*Helianthus tuberosus* L.) plantlets under NaCl stress. We also examined HtP5CS, HtOAT and HtPDH enzyme activities and gene expression patterns of putative *HtP5CS1, HtP5CS2, HtOAT, HtPDH1,* and *HtPDH2* genes. The objective of our study was to characterize the proline regulation mechanisms of Jerusalem artichoke, a moderately salt tolerant species, under NaCl stress. Jerusalem artichoke plantlets were observed to accumulate proline in roots, stems and leaves during salt stress. HtP5CS enzyme activities were increased under NaCl stress, while HtOAT and HtPDH activities generally repressed. Transcript levels of *HtP5CS2* increased while transcript levels of *HtOAT*, *HtPDH1* and *HtPDH2* generally decreased in response to NaCl stress. Our results supports that for Jerusalem artichoke, proline synthesis under salt stress is mainly through the Glu pathway, and *HtP5CS2* is predominant in this process while *HtOAT* plays a less important role. Both *HtPDH* genes may function in proline degradation.

## Introduction

Salt stress is a serious abiotic stresses in agricultural production [1]. Soil salinity has been intensified more by global climate change due to the increased temperature in the future. There is the urgent need to identify and use salinity tolerant plants. Since plants can’t move, many have evolved sophisticated mechanisms to adapt to undesirable environments. Osmotic regulation is an important mechanism for plant cellular homeostasis in saline conditions. Under salt stress, plants accumulate several compatible solutes in the cytosol, such as polyols, betaine, trehalsose, ectoine, proline and others [2]. As a most common osmolyte for osmoprotection, proline has been extensively researched.

Two proline biosynthetic pathways have been elucidated in higher plants: the glutamate and ornithine pathways [3], [4]. In the glutamate (Glu) pathway, glutamate is first catalyzed by P5CS (Pyrroline-5-carboxylate synthetase) to generate GSA (Glutamate-γ-semialdehyde), which equilibrates with P5C (Pyrroline-5-carboxylate). Proline is produced from P5C when it is reduced by P5CR (Pyrroline-5-carboxylate reductase). In this pathway, P5CS is identified to be the rate-limiting enzyme [5]. In the ornithine (Orn) pathway, the precursor ornithine is transaminated to P5C by δ-OAT (Orn-δ-aminotransferase), which is considered to be the key enzyme in this pathway. In general, proline biosynthesis is considered to be mainly through Glu pathway under stress conditions while the Orn pathway is involved in seedling development [6]. Proline catabolism is mainly through proline dehydrogenase or oxidase (PDH) and P5C dehydrogenase, which produce P5C and Glu, respectively [7].

Several reports reveal that overexpression of *P5CS* results in an increased proline level as well as osmotolerance in transgenic plants [8]–[11]. However, the relationship between proline accumulation and stress tolerance is not always clear. Proline content was not consistent with salt tolerance in barley [12]. In addition, excess accumulation of proline was toxic in *pdh* knockout mutant plants [13]. There are also indications that *AtPDH* is induced by proline while repressed by osmotic stress [14]. Therefore, the relationship between proline accumulation and stress tolerance is unclear for individual plant species. Different plants may use individual strategies to fine-tune gene regulation under stress.

Previously, we demonstrated that proline content was significantly increased under NaCl treatments in the roots of Jerusalem artichoke (*Helianthus tuberosus* L.) [15]. Jerusalem artichoke, a species of sunflower, is considered as moderately salt tolerant [16]. While proline levels in plants are determined by biosynthesis, catabolism, and transport, the proline biosynthesis and degradation pathways are not clear for Jerusalem artichoke. In this paper, we cloned several putative proline-related genes in order to further investigate proline metabolism under salinity stress in Jerusalem artichoke, a multifunctional crop in food or potential bioethanol fuel [17]. Based on proline tissue distribution, enzyme activities and gene expression profiles, the objective of our investigation was to characterize proline regulation mechanisms under NaCl stress.

## Materials and Methods

### Plant Material, Growth Condition and Stress Treatments

Jerusalem artichoke (Nanyu No.1) was vegetatively propagated as previously described [15]. Briefly, tuber slices with one bud (about 2.0 g) were rinsed thoroughly with deionized water after surface sterilization. The tuber slices were covered with moist acid-washed quartz sand, supplied with 1/2 Hoagland solution and then sprouted for seven days in plastic containers [18]. Plantlets of uniform size (about 20.0 cm) with three fully-extended leaves and one expanding leaf were chosen for salinity treatments after about one month. Salinity treatments included 500 ml 1/2 Hoagland solution plus 50 mM, 100 mM or 200 mM NaCl, individually. Control plantlets received only 500 ml 1/2 Hoagland solution. The plants were grown in the greenhouse with a light intensity of 392∼415 µmol m^−2^ s^−1^ during a 12-h light at 25°C and 12-h dark at 18°C. Tissue samples were harvested and immediately dipped in liquid nitrogen and stored at −80°C until use (for gene expression analysis). To study relative gene expression in time-course experiments, roots, stems, and leaves were collected at 0, 1, 4 and 12 h under 0 or 100 mM NaCl treatments. For NaCl dosage experiments, samples were supplied with 500 ml 1/2 Hoagland solution including 0, 50, 100 or 200 mM NaCl treatments and harvested 12 h after treatments. Each treatment had three biological replications.

### Proline Content Determination

Fresh roots, stems, and leaves were collected 0, 12, 24, 48, and 72 h after NaCl treatments (0 or 100 mM). Proline content was determined by ninhydrin assay at A_520_ nm according to the method of Bates *et al.* [19].

### P5CS, OAT and PDH Enzymes Activity Assay

The above tissue samples for proline content measurements were also used for the following enzyme activity assays.

For the P5CS enzyme activity assay, protein was extracted according to Hayzer *et al*. [20] with minor modification. Briefly, 1.0 g sample was homogenized in 3.0 ml of ice-cold extraction buffer containing 0.5 M Tris-HCl (pH 7.5), 10 mM MgC1_2_, 2 mM PMSF (phenylmethylsulfonyl ﬂuoride), and 2% PVP (polyvinyl-pyrrolidone). The homogenate was centrifuged at 20,000 g for 20 min (4°C). The supernatant was collected as crude extract. The reaction was initiated by adding 1 ml crude extract into 3 ml reaction medium containing 50 mM Tris-HCl (pH 7.0), 20 mM MgCl_2_, 10 mM ATP, l00 mM Hydroxamate-HCl, 50 mM L-glutamate. The reaction was stopped after 30 min at 37°C by adding 3 ml terminating solution containing FeCl_3_ (5%, w/v) and trichloroacetic acid (12%, w/v) in 5 M HCl. The protein was removed by centrifugation and the supernatant was recorded at A_535_ nm against the tube without ATP as control. The enzyme activity expressed as U/g FW. One U was defined for the enzyme content producing 1 µmol γ-gutamine in 1 min.

The enzyme activity of OAT in each sample was determined by homogenizing 1.0 g plant tissue in 3.0 ml 50 mM ice-cold phosphate buffer (pH 8.0) containing 1 mM DTT (dithiothreitol). The supernatant was collected for the enzyme assay after centrifugation at 7000 g for 20 min (4°C). The enzyme activities were determined by ninhydrin method described by *Kim et al.* [21]. One U of OAT activity represented 1 mmol P5C produced per hour.

PDH activity was assayed according to Lutts *et al.* [22]. Plant tissue (1.0 g) was homogenized in 3.0 ml ice-cold extraction buffer containing 50 mM Tris-HCl (pH 7.4), 10 mM β - mercaptoethanol, 7 mM MgCl_2_, 3 mM EDTA (ethylene diamine tetraacetic acid), and 0.6 M KCl. The homogenate was centrifuged at 12,000 g for 20 min (4°C) and the supernatant was collected as crude extract. For activity determination, 0.2 ml crude extract was added into reaction mixture buffer containing 0.15 mM NaCO_3_-HCl (pH 10.3), 15 mM L-proline and 1.5 mM NAD^+^. A_340_ was recorded at 25°C. The result was expressed as U/g FW. One U represented the reduction value of 0.01 A_340_ per minute.

### *RNA* Isolation and *cDNA* Synthesis

Total RNA was extracted using E.Z.N.A. Plant RNA Kit (OMEGA, R6827-02). In order to remove residual genomic DNA, 1 µg total RNA was treated with Rnase-Free Dnase Set (OMEGA, E1091-02). PrimeScript RT-PCR kit (Takara, D6110A) was used for cDNA synthesis following the instructions of the manufacturer.

### Cloning cDNA for *P5CS*, *OAT*, and *PDH*

Gene cloning proceeded according to Liang *et al*. [23]. Briefly, Jerusalem artichoke ESTs (Expressed Sequence Tags) were downloaded from NCBI website. A database of more than 40,000 retrieved ESTs was created in Bioedit software (Tom Hall, 7.0.9). P5CS, OAT and PDH cDNA sequences from Arabidopsis and rice were used to Localblast this EST database. Assembled by high similarity EST sequences, candidate genes were cloned for further bioinformatic analysis. All five Jerusalem artichoke gene sequences were deposited into the NCBI GeneBank. The accession numbers for *HtP5CS1*, *HtP5CS2*, *HtOAT*, *HtPDH1* and *HtPDH2* in the genebank are KC700610, KC700611, KC700612, KC700613, and KC700614, respectively.

### Bioinformatic Analysis

The protein sequences of target genes from Genebank were used for sequence alignment analysis by the ClustalW method in the MegAlign program (DNASTAR, Inc., Madison, WI). A phylogenetic tree was generated based on the ClustalW protein alignment analysis using a Neighbor-Joining method in the MEGA 4 program. The following sequences with corresponding accession numbers were used for bioinformatics analysis: *AtP5CS1* (NM_129539), *AtP5CS2* (NM_115419.4); *OsP5CS1* (D49714.1), *OsP5CS2* (NM_001051337); *PvP5CS1* (EU340347), *PvP5CS2* (EU407263); *SbP5CS1* (GQ377719), *SbP5CS2* (GQ377720); *AtOAT* (NM_123987.3); *BnOAT* (EU375566.1); *GmOAT* (NM_001250221.1); *MtOAT* (AJ278819); *NtOAT* (ADM47437); *AtPDH1* (NM_113981.5), *AtPDH2* (NM_123232.2); *MsPDH* (AY556386); *NtPDH1* (AY639145.1); *NtPDH2* (AY639146.1).

### Quantitative PCR (qPCR) Assay and Data Analysis

Quantitative PCR was performed using SYBR Premix EX Taq (Takara, DRR420A) in the ABI 7500 Real Time PCR System according to the manufacturer’s instructions. The 20 µl reaction system contained 10 µl SYBR Premix Ex Taq, 0.4µl each of 10 µM primers, 0.4µl ROX Reference DyeII (50×), 2.0 µl DNA template, and 6.8 µl dd H_2_O. The program consisted of initial denaturation at 95C for 30 s, followed by 40 cycles of 95C for 3 s, annealing for 34 s at 60 C. The dissociation stage includes 95C for 15 s, 60C for 1 min and 95C for 15 s. The Jerusalem artichoke housekeeping gene actin was used for normalization of the amount of cDNA in each qPCR reaction. Since the deviation error of amplification efficiency between target genes and reference gene were less than 10% according to our trial experiments, data were processed using the method of 2^− ΔΔCT^ according to Livak *et al*. [24]. For gene transcript level analysis on time-course or salt dosage experiments, gene transcript level in each treatment sample was further normalized to corresponding time-point control sample to avoid circadian rhythm interference. Fold changes were used for statistical analysis.

### Primers

Primers for gene cloning and qPCR are listed in Table S1 and Table S2.

### Statistical Analysis

Data were analyzed by Microsoft Excel 2003 (Microsoft Corp., Redmond, WA, USA) and SPSS 16.0 (SPSS Corp., Chicago, IL, USA). Each data point shown in the figures is the mean of three biological replications. For each individual sample in real-time PCR experiments, three further technical replications were conducted. Internal significant differences among treatments were statistically evaluated by One-Way ANOVA using Duncan’s t-test. Figures were created using SigmaPlot 10.0 (Systat Software, Inc. Germany).

## Results

### NaCl Stress Enhanced Proline Accumulation in Roots, Stems and Leaves

**Figure 1:**
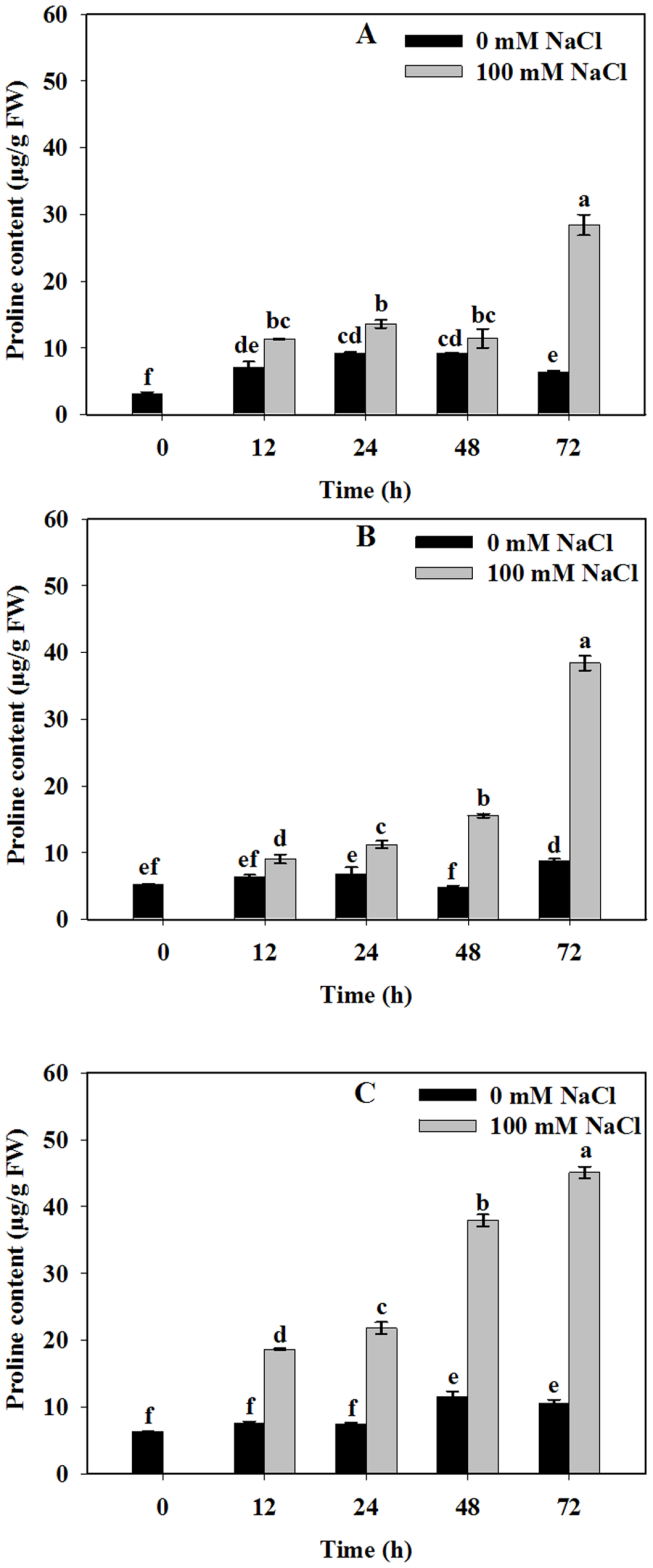
Effect of NaCl stress on proline accumulation of Jerusalem artichoke in roots (A), stems (B) and leaves (C). 30-day-old Jerusalem artichoke plantlets were treated with 0 or 100 mM NaCl (see Materials and Methods) for 12, 24, 48 and 72 h, respectively. All treatments had three biological replicates. Proline contents were measured by ninhydrin assay at A_520_ nm. Values represent means ± SE of three independent experiments. Significant differences (P≤0.05) between treatments are indicated by different letters.

To determine the relationship between salt tolerance and proline content in Jerusalem artichoke, we first examined whether salinity stress would enhance the proline levels in different tissues. Our previous experiments indicated that proline level gradually increased with salt concentrations in roots [15]. Since Jerusalem artichoke plants are mostly planted in shoreland or saline land in China, salt concentrations in the soil are usually below 150 mM. As a moderately salt-tolerant species, Jerusalem artichoke can accomplish its life cycle under 100 mM NaCl treatment [25]. **Fig. 1** shows a significant increase in proline content could be observed in roots, stems and leaves treated with 100 mM NaCl solution during the initial 72 h period. Proline accumulated in roots, stems and leaves were 28.42, 38.39, 45.10 µg/g at 72 h, respectively, which were three fold higher than each control. Proline accumulated more in leaf samples than roots or stems at the same time point.

### NaCl Stress Increased HtP5CS Activities while Repressed HtOAT and HtPDH Activities

**Figure 2:**
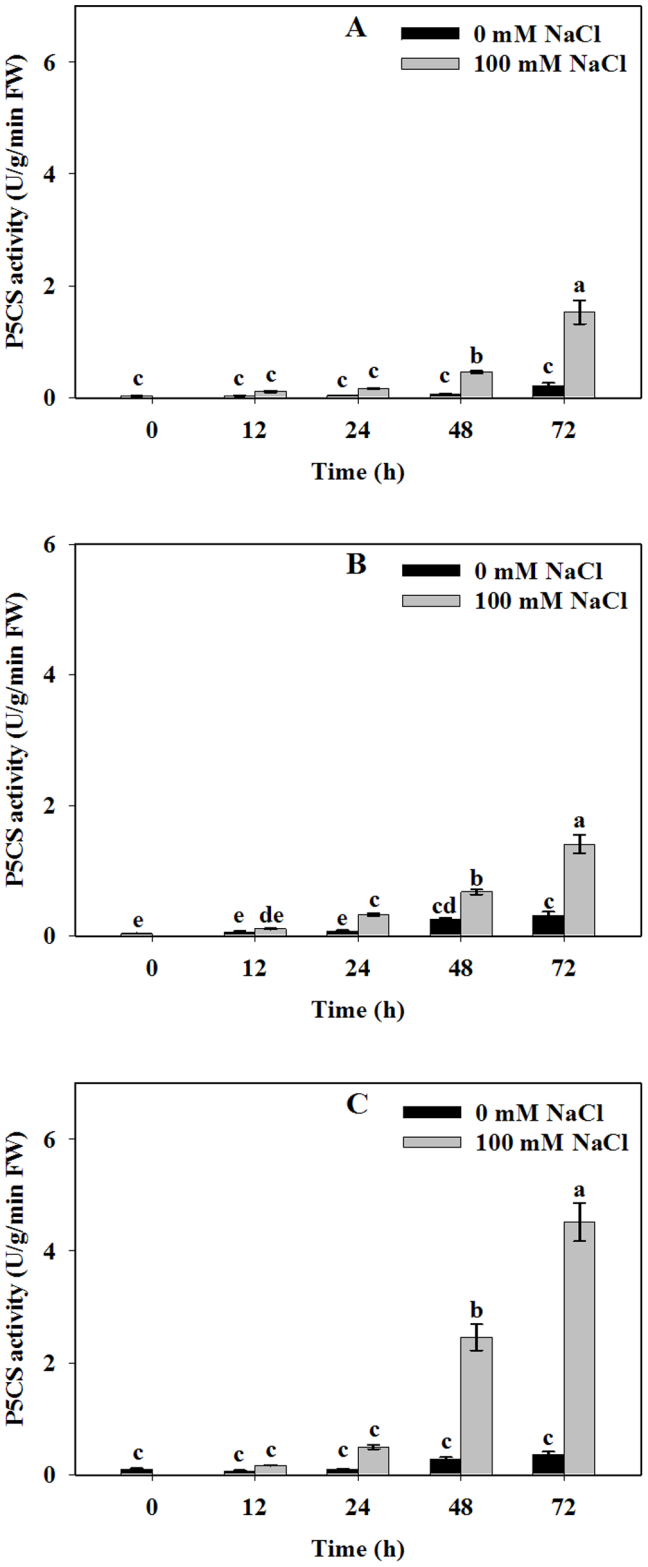
Effect of NaCl stress on *P5CS* activities of Jerusalem artichoke in roots (A), stems (B) and leaves (C). 30-day-old Jerusalem artichoke plantlets were treated with 0 or 100 mM NaCl (see Materials and Methods) for 12, 24, 48 and 72 h. All treatments had three biological replicates. P5CS activities were measured by hydroxylamine hydrochloride assay at A_535_ nm (see Materials and Methods). Values represent means ± SE of three independent experiments. Significant differences (P≤0.05) between treatments are indicated by different letters.

Recent evidence indicates that proline accumulation is mainly initiated by *de novo* synthesis [26]. To explore the reason why proline was accumulated in different tissues, enzyme activities in proline metabolism pathways were investigated. HtP5CS activity increased in response to NaCl stress in all three tissues. A significant increase of HtP5CS activity could be found in stems of Jerusalem artichoke after 24 h NaCl exposure, while for roots and leaves, significant changes could be found after 48 h stress treatment. Leaf samples showed the highest HtP5CS enzyme activity under 72 h NaCl treatments **(** **Fig. 2** **)**. HtP5CS enzyme activities in all tissues were consistent with proline levels which implies that proline biosynthesis mainly originates from the Glu pathway under salt stress conditions.

**Figure 3:**
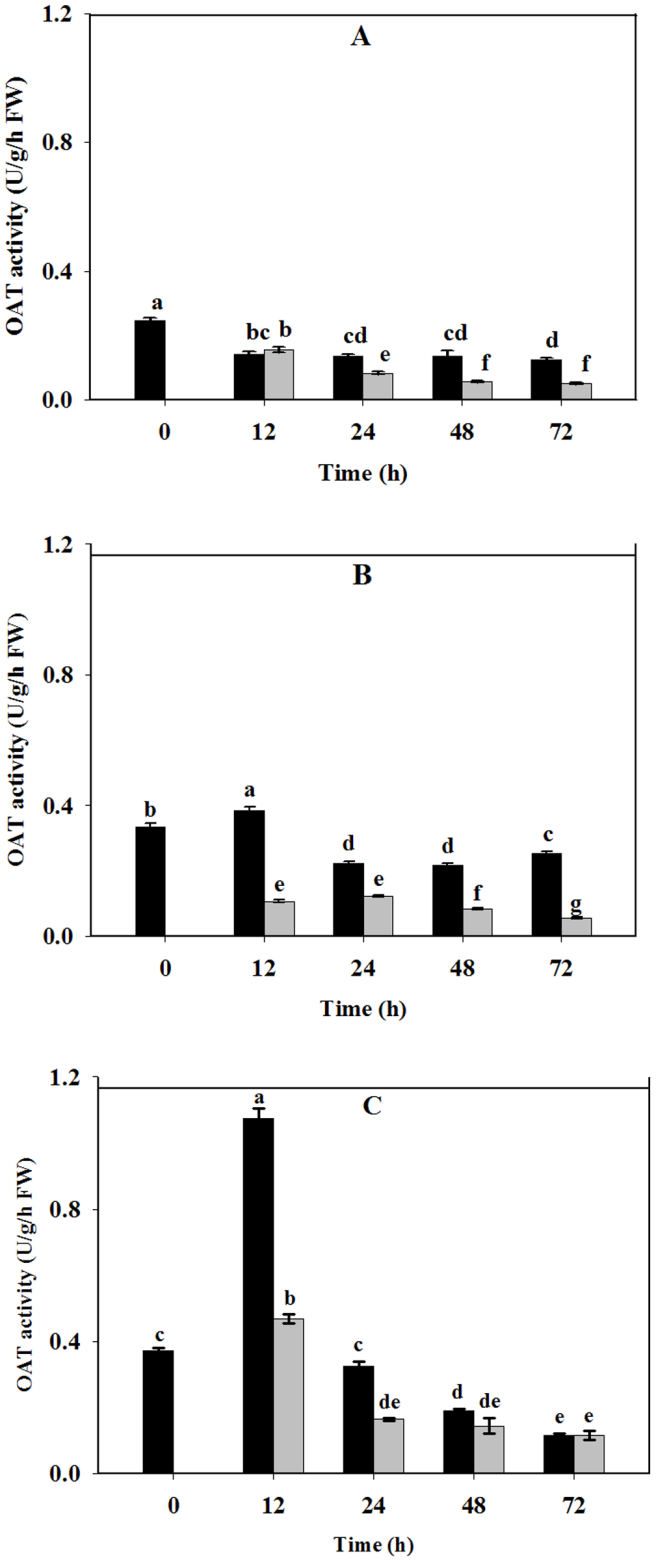
Effect of NaCl stress on δ-OAT activities of Jerusalem artichoke in roots (A), stems (B) and leaves (C). 30-day-old Jerusalem artichoke plantlets were treated with 0 or 100 mM NaCl (see Materials and Methods) for 12, 24, 48 and 72 h. All treatments had three biological replicates. OAT activities were measured by ninhydrin method (see Materials and Methods). Values represent means ± SE of three independent experiments. Significant differences (P≤0.05) between treatments are indicated by different letters.

In contrast to HtP5CS enzyme activities, HtOAT enzyme activities showed the opposite trend. HtOAT activities were generally inhibited under NaCl stress duration from 12 to 72 h. NaCl stress decreased HtOAT activities significantly in stems and leaves of Jerusalem artichoke after 12 h stress treatment. In roots, significant decrease happened after 24 h exposure to stress **(** **Fig. 3** **)**.

**Figure 4:**
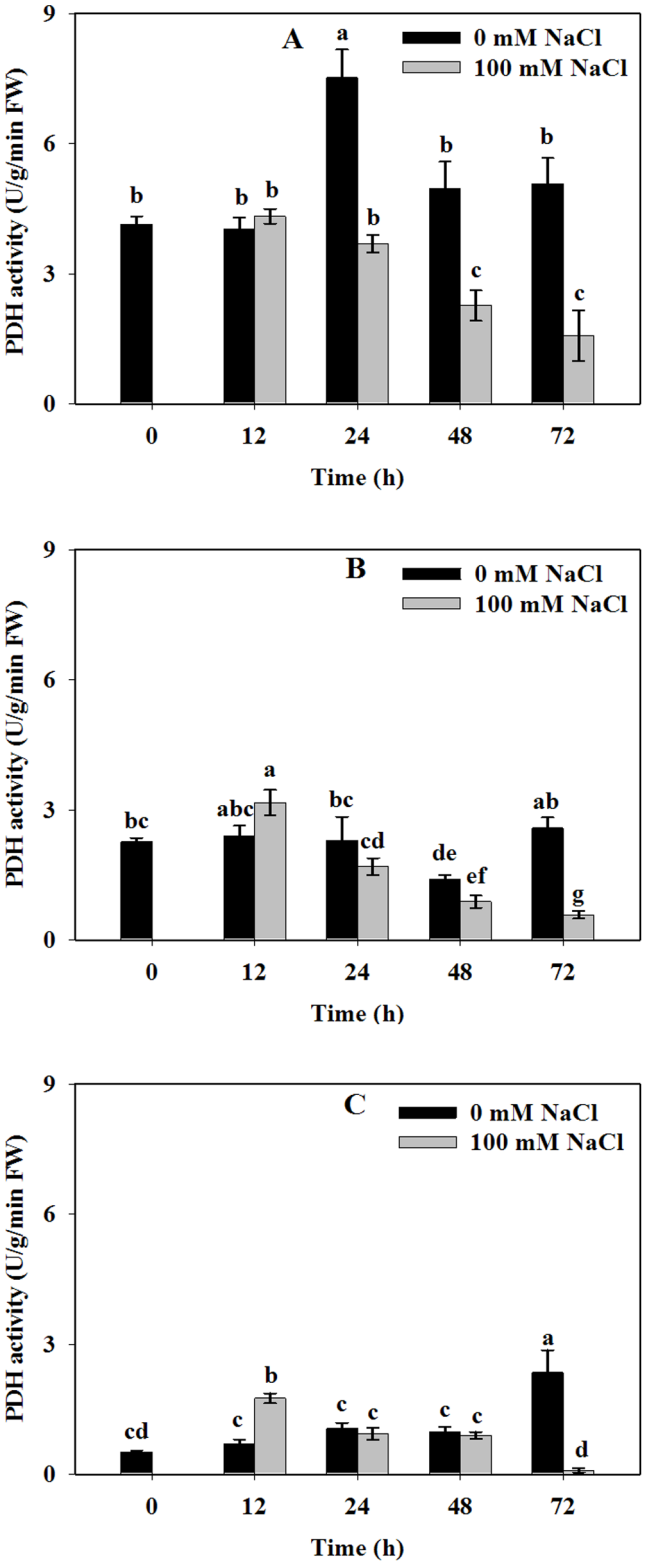
Effect of NaCl stress on PDH activity of Jerusalem artichoke in roots (A), stems (B) and leaves (C). 30-day-old Jerusalem artichoke plantlets were treated with 0 or 100 mM NaCl (see Materials and Methods) for 12, 24, 48 and 72 h. All treatments had three biological replicates. OAT activities were measured by spectrophotometry at A_340_ at 25°C (see Materials and Methods). Values represent means ± SE of three independent experiments. Significant differences (P≤0.05) between treatments are indicated by different letters.

PDH enzyme plays a key role in proline catabolism. Our results demonstrated that salinity stress gradually decreased the HtPDH activities in roots and stems of Jerusalem artichoke at 24 h while activities stayed constant in leaves except at 72 h **(** **Fig. 4** **)**. HtPDH activities were relative lower in the leaves, compared to that of roots and stems regardless of NaCl stress.

It was a little surprising that proline content is increased in all measured tissues while HtP5CS enzyme activities were almost the same in all treatments of NaCl at 12 h. We hypothesized that our enzyme activity measurement method may not be responsive enough to detect rapid changes as a result of external stimuli. To provide further information about proline metabolism in Jerusalem artichoke, several possible proline-metabolism genes were then cloned.

### Bioinformatics Analysis of Five Proline-metabolism Genes

In contrast to yeast and animal system, plant genes always form a multimember gene family. Family members may play redundant roles or have function differentiations. According to published Arabidopsis and rice information, we postulated at least two *P5CS* and *PDH* isoforms in Jerusalem artichoke. Gene information of *α-OAT*s were reported in bacterium, while only *δ-OAT*s were found in higher plants [27], therefore we only studied *δ-OAT* in this paper.

Using Arabidopsis and rice P5CS nucleotide sequences to Blastn the Jerusalem artichoke EST database, twelve ESTs with high similarities were retrieved, that is,125441546, 125423128, 125430151, 125443641, 125446643, 125448887, 125449956, 12550355, 125429963, 125439651, 125439845 and 125442867. Assembled by SeqMan™II(DNASTAR), two contigs were formed. The first Contig was 2516 bp length and the deduced protein showed high identity to reported P5CS sequences. Primers were designed to amplify contig 1 (using primer 1, Table S1), and the results revealed a 2496 bp cDNA harboring a full-length putative P5CS (2154 bp) based on the alignment with other P5CS sequences. Contig 2 only had partial CDS sequences, so efforts were made to search against other Compositae crop’s EST database, such as sunflower. It was found that 58736240 in sunflower EST database showed high similarity with the front part of contig 2 (http://compbio.dfci.harvard.edu/cgi-bin/tgi/gimain.pl?gudb=f_arundinacea), and 113187185 from silverleaf sunflower (*Helianthus argophyllus* L.) EST database hits to the back region, so these two sequences were then assembled to form contig 2 to design the primer pair. Sequenced after PCR amplification (using primer 2, Table S1), a 2257 bp sequence was obtained without full-length CDS. A 3′-end RACE PCR (Clontech, Cat.No. 634923, USA) was carried out to extend the 3′-end (using primer 3, Table S1). By using this method, contig 2 was successfully prolonged and a full-length sequence of 2178 bp CDS was amplified (using primer 4, Table S1). According to the similarity with other P5CSs, contig 1 was named as HtP5CS1, whose deduced amino sequence showed highest similarity with PvP5CS1 (79.1%), and contig 2 was named as HtP5CS2, with 72.9% identity with PvP5CS2.

Using *AtOAT* and *BnOAT* CDS sequences to Blastn Jerusalem artichoke EST database, we found nine ESTs with high similarities, that is, 125422045, 125426820, 125427805, 125431428, 125437709, 125439438, 125440189, 125443871 and 125450877. Those ESTs were then assembled into a 1265 bp template. Using Ht cDNA as template, a putative *HtOAT* with a product of 1146 bp length was amplified (using primer 5, Table S1), however, without a complete CDS. The sequence of this putative *HtOAT* was compared with other compositae plants. We retrieved 211619016 from the sunflower EST database, 113237927 from the serpentine sunflower (H*elianthus exilis* L.) EST database, and 90489792 from the plains sunflower (*Helianthus petiolaris* L.) EST database. The retrieved sequences were used for assembling the putative partial CDS into a 1681 bp sequence. Sequenced after PCR (using primer 6, Table S1), a sequence including a full-length sequence of 1410 bp was obtained, whose deduced amino acid sequences shared 77.2% similarity with NtOAT.

Using *BnPDH* to Blastn against Ht EST database, we identified thirteen ESTs with high similarities and assembled these into two contigs (125424918, 125425174, 125425617, 125427731, 125428176, 125437754, 125456916, 125457059, 125422040, 125432267, 125434747, 125459104 and 125450265). Contig 1 with 1867 bp length was used for primer design. Using Ht cDNA as template, a 1618 bp product was amplified (using primer 7, Table S1), which was found to include a full-length CDS (1497 bp). Contig1 had 57.7% similarity with NtPDH1 and was named as HtPDH1. For contig 2, eight sunflower ESTs were retrieved (22314834, 22396411, 90454266, 90456587, 90457722, 90463063, 90464320 and 90454869). These ESTs were assembled to form a contig2. PCR amplification (using primer 8, Table S1) and sequencing identified that a 1581 bp sequence existed and that it appeared to be a full-length putative CDS. Contig2 showed highest similarity with NtPDH2 (58.4%), and was named as HtPDH2.

By using these methods, we acquired five proline-metabolism genes: two *P5CS*s, one *δ-OAT* and two *PDH*s. The deduced protein sequence of putative HtP5CS1 showed an 83% identity to *Actinidia deliciosa* P5CS, which has been reported by Walton *et al* [28]. For HtP5CS2, the similarity was 76% to that of predicted *A.deliciosa* P5CS. Putative HtOAT showed 76% similarity with NtOAT whose function is still not confirmed, and 72.7% identity with AtOAT which involved in proline synthesis [29]. HtPDH1 showed 59% similarity to *N.tabacum* Cig1 (Cytokinin-induced gene) which functioned in proline degradation [30]. Also, HtPDH2 showed 60% similar to *N.tabacum* proline oxidase/dehydrogenase 2 which has not been well illustrated until now.

Compared with other duplicated P5CS isomers, sequences analysis indicated that the putative HtP5CS1 and HtP5CS2 had several conserved regions including a putative ATP binding site, conserved Leu Zipper, conserved Glu-5-kinase domain, NAD(P)H binding domain, putative Leu domain, and conserved GSA-DH domain which has been reported in sorghum [31] (Fig. S1). In the deduced HtOAT protein, from amino site 226 to 294, was a putative pysidoxal phosphate-binding site which has been illustrated in Arabidopsis [3] (Fig. S2). A proline dehydrogenase domain was also found in HtPDH1 and HtPDH2, which is similar in tobacco [32] (Supplement Fig. 3).

### Expression Profiles of Five Proline-metabolism Genes under Salt Treatments

To link proline-metabolism genes with physiological processes, transcript levels of each Jerusalem artichoke genes were studied in different tissues or at different developmental stages or stress conditions. Relative transcript levels of each gene in different tissues and at different developmental stages were quantified and compared with the level of control roots (arbitrary selection) to demonstrate the relative transcript levels of the same gene.

**Figure 5:**
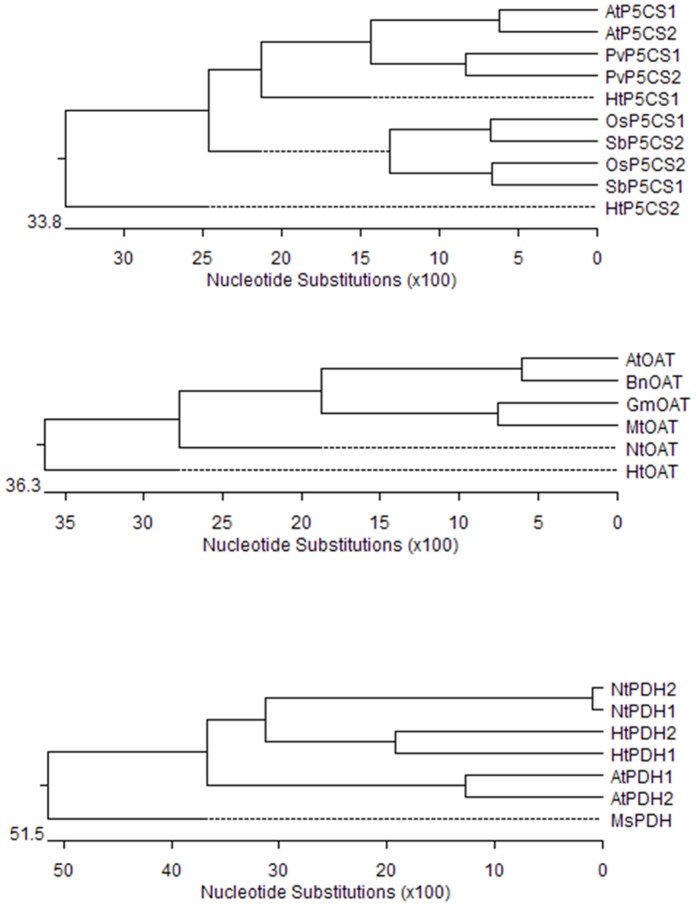
Phylogenetic analysis of *HtP5CS, HtOAT and HtPDH*. Protein sequences which had been reported were selected for sequence alignment. Sequence alignment analysis was performed using the ClustalW method in the Meglign program (DNASTAR, Inc., Madison, WI, USA). These genes’ accession numbers are : AtP5CS1 (NM_129539) and AtP5CS2 (NM_115419.4) in *Arabidopsis*; OsP5CS1 (D49714.1) and OsP5CS2 (NM_001051337) in rice (*Oryza sativa*); PvP5CS1 (EU340347), PvP5CS2 (EU407263) in common bean (*Phaseolus vulgaris*); SbP5CS1(GQ377719) and SbP5CS2(GQ377720) in sorghum (*Sorghum bicolor*); AtOAT (NM_123987.3) in *Arabidopsis*; BnOAT (EU375566.1) in rapeseed (*Brassica Napus*); GmOAT (NM_001250221.1) in soybean (*Glycine max*); MtOAT (AJ278819) in alfalfa (*Medicago truncatula*); NtOAT (ADM47437) in tobacco (*Nicotiana tabacum*);AtPDH1 (NM_113981.5) and AtPDH2 (NM_123232.2) in *Arabidopsis*; MsPDH (AY556386.1) in alfalfa (*Medicago sativa*); NtPDH1 (AY639145.1) and NtPDH2 (AY639146.1) in tobacco (*Nicotiana tabacum*).

**Figure 6:**
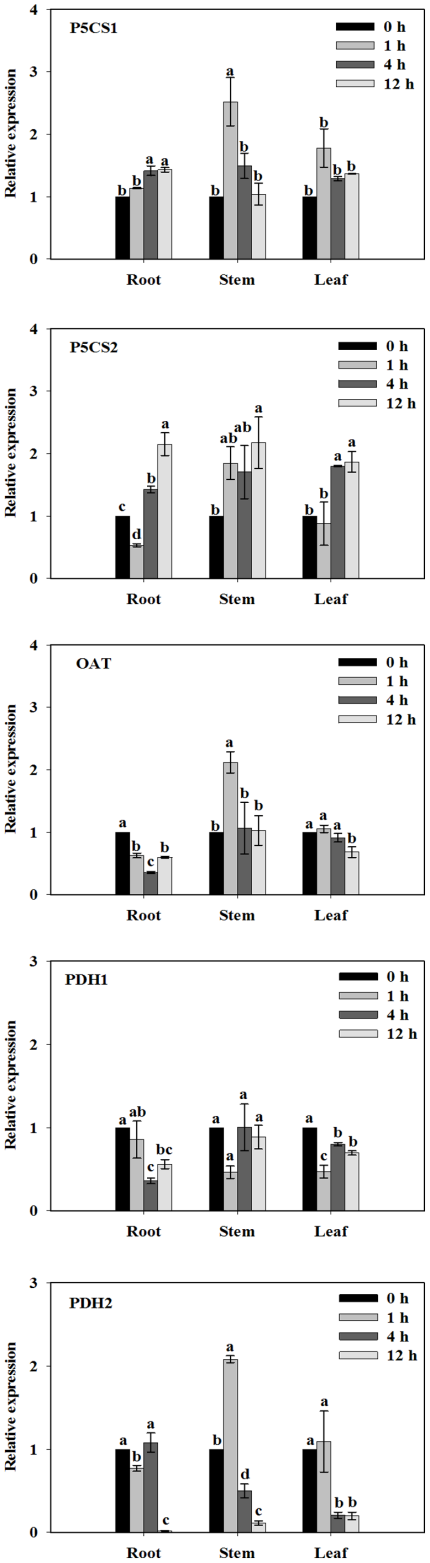
Expression profiles of *HtP5CS1, HtP5CS2, HtOAT, HtPDH1, and HtPDH2* from roots, stems, and leaves under 100 mM NaCl stress in the time-course experiments. 30-day-old Jerusalem artichoke plantlets were treated with 0 or 100 mM NaCl (see Materials and Methods) for 1, 4 and 12 h individually. All treatments had three biological replicates. Total RNA was extracted from roots, stems and leaves for quantitative PCR (RT-qPCR) analysis. Transcript levels were first normalized to the level of a housekeeping gene actin (*HtActin*). The normalized transcript levels were then compared between treatment (100 mM NaCl) and control (0 mM NaCl) to calculate a fold change. Values represent means ± SE of three independent experiments. Significant differences (P≤0.05) between treatments are indicated by different letters.

Gene expression of *HtP5CS1* only changed significantly in roots after 4 h treatment. Transcript levels of *HtP5CS1* were increased in stems after 1 h exposed to 100 mM NaCl stress and decreased after that. However, the expression level of *HtP5CS1* in the leaves was almost as same as the control (**Fig. 5** **and** **6**). Generally speaking, expression pattern of *HtP5CS2* showed a similar trend with proline accumulation profiles in all three tissues examined. Transcript level of *HtP5CS2* was initially induced in roots and leaves after 4 h treatment and reached the highest level at 12 h. In stems, expression levels of *HtP5CS2* were only increased at 12 h (**Fig. 6**).

NaCl stress mildly inhibited *HtOAT* expression in roots within the 12 h experiment period while in leaves a significant decrease appeared only after 12 h exposed to 100 mM NaCl treatment. In stems, the expression level of *HtOAT* was raised after 1 h stress treatment, however, expression decreased to the control level after that (**Fig. 6**).

NaCl stress down-regulated the expression of *HtPDH1* in roots and leaves while no significant change could be found in stems compared to the control (**Fig. 6**). Expression levels of *HtPDH2* in all three tissues were gradually decreased according to our experiments. However, drastic changes were only observed at 4-h stems and leaves or 12-h roots.

## Discussion

### NaCl Stress Promoted Proline Accumulation by Activating Proline Synthesis and Inhibiting Proline Catabolism Simultaneously

Previously, Newton *et al* [25] reported that Jerusalem artichoke was a moderately salt tolerant crop when EC_e_ was 7.5 dS m^−1^ in soil. In the present study, we simulated a series of NaCl-induced stresses ranging from 3.48 dS m^−1^ (50 mM NaCl+1/2 Hoagland) to 9.82 dS m^−1^(200 mM NaCl+1/2 Hoagland). Our results indicated that proline content was enhanced in Jerusalem artichoke roots, stems and leaves based on time-course experiments under 100 mM NaCl stress (EC_e_ = 6.26 dS m^−1^) (Fig. 1). The same finding was reported in canola [33] and rice callus tissue [9]. By comparing different cultivars of sunflower in response to salt stress, Shahbaz *et al* [37] also found that salt tolerant cultivars exhibited greater proline accumulation. Proline content was observed higher in leaves in this study, which was same with previous study in green gram [34], mulberry [35] and canola [36]. It has been suggested that leaves accumulate more proline in order to maintain chlorophyll level and cell turgor to protect photosynthetic activity under salt stress [38]. This is consistent with our results indicating that proline accumulation was greatest in the leaves of stressed plants.

Tissue accumulations of proline under stress may result mainly from de-novo biosynthesis since proline content was increased in all three tissues compared to the control, similar to previous results [39]. To explore the mechanism behind this, we measured the rate-limiting enzyme activities in the two primary proline synthetic pathways respectively. Our results revealed that HtP5CS activities were unambiguously stimulated while HtOAT activities were slightly repressed after the plantlets were exposed to NaCl treatments (Fig 2, Fig. 3).In general, we found that tissues that had higher HtP5CS enzyme activities accumulated more proline. In our study, HtOAT enzyme activities were lower in all three tissues under salt treatment. However, Wang *et al* [40] claimed that in *S.amara,* a typical halophyte, δ-OAT activity was also enhanced, contributing to proline synthesis. We inferred that proline synthesis under stress is likely species-dependent. For Jerusalem artichoke, the Glu pathway was predominant while the Orn pathway played a minor role, if any, under our stress conditions. Our findings were also consistent with Delauney *et al* [41].

Interestingly, PDH activity was repressed under NaCl stress (Fig. 4), which had been illustrated previously in rice [42]. Moreover, our results showed that PDH activity was higher in roots than in leaves, which was also accordance with the study in rice [43]. Higher P5CS enzyme activity and lower PDH activity may both contribute to the highest proline content in leaves. These results indicate that proline accumulates in Jerusalem artichoke plantlets under NaCl stress by activation of proline synthesis through the glu pathway, while at the same time, repression of degradation.

### Sequences Analysis of Five Putative Proline-metabolism Genes in Jerusalem Artichoke Revealed High Similarity to Identified Genes

Since at least two P5CS genes, one δ*-*OAT and two PDH genes were already characterized in the plants, to better illustrate the proline metabolism in Jersalem artichoke, we cloned several putative proline metabolism genes through EST alignment according to the reported nucleotide sequences. Deduced amino sequences of the five cloned genes all showed high similarities to the reported genes. According to phylogenetic analysis, the putative HtP5CS1 protein shared the highest similarity with PvP5CS1 (79.1%), and HtP5CS2 showed 73.7% similarity with PvP5CS1. HtOAT showed the highest homology with NtOAT (77.2%). Two HtPDHs were grouped with NtPDHs. The highest similarity of HtPDH1 could be found with NtPDH2 (57.9%), and HtPDH2 shared 58.4% homolog with NtPDH2. By comparisons of function-identified protein sequences, the deduced protein sequences showed that both HtP5CS1 and HtP5CS2 had a conserved AA_Kinase and Aldedh domains and an aminotran_3 was found in HtOAT (http://pfam.sanger.ac.uk/). For HtPDH1 and HtPDH2, a pro_dh domain exists. Via alignment with other species, all five genes have conserved domains, such as ATP-binding domain in P5CS genes, pyridoxal phosphate-binding in OAT gene and a proline dehydrogenase domain in the PDH gene. Sequences analysis indicated that our cloned genes could be the actual proline-metabolism genes.

### Transcript Levels of *HtP5CS2* were Increased while Expression Levels of *HtOAT*, *HtPDH1* and *HtPDH2* were Generally Decreased in Response to NaCl Stress

Since enzyme activity measurement methods could not promptly reflect an immediate change, we examined expression patterns in five genes in the proline metabolic pathway. Our results revealed that the induction of *HtP5CS2* was initiated within 12 h by NaCl stress in different tissues. Also, the same trend was found when the plantlets were exposed to different NaCl dosages in 12-h treatments (Fig. S4). In our study, NaCl stress did not repress relative expression of *HtP5CS1* (Fig. 6), however, the change of gene expression level was much less than that of *HtP5CS2*. In cactus pear (*Opuntia streptacantha* L.), Silva-Ortega *et al.* [38] found that the transcript level of *OsP5CS* (accession number:D49714.1, higher similarity to *HtP5CS1*) was increased with NaCl concentrations at the 9th day after treatment. Szekely *et al* [44] reported that knock-out Arabidopsis *p5cs1* mutant plants were hypersensitive to salinity, whereas *AtP5CS2* was essential for seedling development. The results presented in the study indicated that for Jerusalem artichoke, both P5CS1 and P5CS2 in Glu pathway may function synergistically. *HtP5CS2* seemed to play predominant roles under salt stress based on its constantly increasing expression. Nevertheless, our results were not completely in line with Armengaud *et al*. [6], who indicated that *MtP5CS2* was more important in the Glu pathway in leaves while the Orn pathway was predominant in roots for proline synthesis under salt stress.

In this present study, δ-OAT activity was repressed by NaCl stress. Relative expression levels of *δ-OAT* also showed a close association with enzyme activity changes. In fact, expression levels of *δ-OAT* in all tissues were almost the same under the 100 mM NaCl treatment, while enzyme activities were slightly decreased. Transcript level of *HtOAT* was greatly induced by 200 mM NaCl treatment while down-regulated by 50 mM salt treatment in roots. In stems, the transcript level of *HtOAT* decreased with higher NaCl concentrations. No significant change of *HtOAT* transcript level could be found in leaves with increased NaCl dosage (Fig S4). By use of T-DNA insertion, Funck *et al* [45] demonstrated that δ-OAT activity was repressed in an *oat* mutant while proline content did not change compared to the wildtype plants under salt stress, therefore, the author proposed that δ-OAT did not contribute to proline synthesis in Arabidopsis under salt stress. Surprisingly, You *et al* [46] observed that overexpression of *OsOAT* in rice had no significant effects on proline accumulation compared to wildtype under high salinity while increasing stress tolerance ability exhibited in the overexpression transgenetic lines was considered to promote the ROS-scavenging capacity. Roosens *et al* [3] reported that *OAT* expression varied with growth period in Arabidopsis under salt stress, whereas the predominant role of the Glu pathway for proline synthesis was identified in adult plants. Based on the previous finding, further research should be carried out in plant developmental stage based on metabolic network to clarify Orn roles under salt stress.

PDH is responsible for catabolizing proline into P5C. As shown in Fig. 4, we found that PDH activities were repressed over time, which was consistent with the expression profile of *HtPDH2* under stress (Fig.6 and Fig S4). Moreover, 50 mM NaCl stress down-regulated the expression of *HtPDH1* in stems. A stress of 200 mM had the greatest effect on the *PDH1* transcript level in roots, while no significant change could be found in stems compared to the control in all salt treatments (Fig. S4). Compared to control, relative expression levels of *HtPDH2* were significantly inhibited under different NaCl stresses in roots, stems and leaves at 12 h. Expression levels of *HtPDH2* in leaves were gradually repressed with higher salt concentrations (Fig. S4). The same trend was found in *Medicago sativa* while Miller *et al* [47] reported that the tanscriptional level of *MsPDH* (accesssion number: AY556386, higher similarity to *HtPDH2*) was NaCl-dosage-dependent and decreased in leaves after 1 h treatment. Funck *et al* [48] found that *AtProDH2* was induced under salinity in vasculature while *AtProDH1* was repressed which suggested that both AtPDHs played non-redundant functions. Compared to *HtPDH1*, transcripts level of *HtPDH2* were generally down-regulated more in all tissues.

### Conclusions

Our experiments indicate that proline is induced in Jersalem artichoke plantlets under salinity stress. More proline is accumulated in leaves as a possible protective metabolic adaptation to prevent leaf tissue from damage under salinity. Proline accumulation is primarily caused by activation of proline synthesis via the glu pathway, while depression of proline degradation also serves to increase proline levels. Using bioinformatic methods to analyze Jerusalem artichoke ESTs, we cloned five putative proline meatabolic-related genes, whose translated amino acid sequences exhibit high similarities to published sequences. Under salinity, proline synthesis activated by *HtP5CS2* may be predominant, while for proline degradation, both HtPDHs may have function. This study supports the hypothesis that proline plays an important role in Jerusalem artichoke osmoprotection. Although extensive work regarding proline metabolism under salt stress has been done, it still remains to be investigated how these five genes in proline metabolism are regulated and how each member in these same gene families plays different roles under stress.

## Supporting Information

Figure S1 **Amino acid sequence alignments of** ***HtP5CS1*** **and** ***HtP5CS2*** **.** Alignment was performed using the ClustalW program. The following sequences with corresponding accession number were used for bioinformatic analysis: *AtP5CS1* (NM_129539), *AtP5CS2* (NM_115419.4); *OsP5CS1* (D49714.1), *OsP5CS2* (NM_001051337); *PvP5CS1* (EU340347), *PvP5CS2* (EU407263); *SbP5CS1* (GQ377719), *SbP5CS2* (GQ377720). Asterisk, semicolon and dot represent the amino that are “identical”, “conserved substitution” and “semi-conserved substitution”, respectively. Boxed sequences showed conserved putative ATP and NAD(P)H-binding sites, GK and GSA-DH domains, and putative Leu-rich regions.
(TIF)

Figure S2 **Amino acid sequence alignments of** ***HtOAT*** **.** Alignment was performed using the ClustalW program. The following sequences with corresponding accession number were used for bioinformatic analysis: *AtOAT* (NM_123987.3); *BnOAT* (EU375566.1); *GmOAT* (NM_001250221.1); *MtOAT* (AJ278819); *NtOAT* (ADM47437). Asterisk, semicolon and dot represent the amino that are “identical”, “conserved substitution” and “semi-conserved substitution”, respectively. Boxed sequences showed putative pyridoxal phosphate-binding domain.
(TIF)

Figure S3 **Amino acid sequence alignments of** ***HtPDH1*** **and** ***HtPDH2*** **.** Alignment was performed using the ClustalW program. The following sequences with corresponding accession number were used for bioinformatic analysis: *AtPDH1* (NM_113981.5), *AtPDH2* (NM_123232.2); *MsPDH* (AY556386.1); *NtPDH1* (AY639145.1), *NtPDH2* (AY639146.1). Asterisk, semicolon and dot represent the amino that are “identical”, “conserved substitution” and “semi-conserved substitution”, respectively. Boxed sequences showed proline dehydrogenase domain.
(TIF)

Figure S4 **Expression profiles of** ***HtP5CS1, HtP5CS2, HtOAT, HtPDH1, and HtPDH2*** **from roots, stems, and leaves under different NaCl dosage stresses at 12 h.** 30-day-old Jerusalem artichoke plantlets were treated with 0, 50, 100 and 200 mM NaCl (see Materials and Methods) for 12 h, respectively. All treatments had three biological replicates. Total RNA was extracted from roots, stems and leaves for quantitative PCR (qPCR) analysis. Transcript levels were first normalized to the level of a control gene actin (*HtActin*). The normalized transcript levels were then compared between treatments (50, 100 and 200 mM NaCl) and control (0 mM NaCl) to obtain a fold change. Values represent means ± SE of three independent experiments. Significant differences (P≤0.05) between treatments are indicated by different letters. Values represent means ± SE of three independent experiments. Significant differences (P≤0.05) between treatments are indicated by different letters.
(TIF)

Table S1 **Primers for gene cloning of** ***HtP5CS1, HtP5CS2, HtOAT, HtPDH1, and HtPDH2*** **.**
(DOC)

Table S2 **Primers for qPCR of** ***HtP5CS1, HtP5CS2, HtOAT, HtPDH1, and HtPDH2*** **.**
(DOC)

## References

[1] Munns R, Tester M (2008) Mechanisms of salinity tolerance. Annual Review of Plant Biology 59: 651–681. 10.1146/annurev.arplant.59.032607.092911 18444910

[2] Hasegawa PM, Bressan RA, Zhu JK, Bohnert HJ (2000) Plant cellular and molecular responses to high salinity. Annual Review of Plant Biology 51: 463–499. 10.1146/annurev.arplant.51.1.463 15012199

[3] Roosens NHCJ, Thu TT, Iskandar HM, Jacobs M (1998) Isolation of the ornithine-δ-aminotransferase cDNA and effect of salt stress on its expression in Arabidopsis thaliana. Plant Physiology 117: 263–271. 9576796 10.1104/pp.117.1.263 PMC35011

[4] Hu CA, Delauney AJ, Verma DP (1992) A bifunctional enzyme (delta^1^-pyrroline-5-carboxylate synthetase) catalyzes the first two steps in proline biosynthesis in plants. Proceedings of the National Academy of Sciences 89: 9354–9358. 10.1073/pnas.89.19.9354 PMC50125 1384052

[5] Yoshiba Y, Kiyosue T, Katagiri T, Ueda H, Mizoguchi T, et al (2002) Correlation between the induction of a gene for Δ^1^-pyrroline-5-carboxylate synthetase and the accumulation of proline in Arabidopsis thaliana under osmotic stress. The Plant Journal 7: 751–760. 10.1046/j.1365-313x.1995.07050751.x 7773306

[6] Armengaud P, Thiery L, Buhot N, Grenier De March G, Savouré A (2004) Transcriptional regulation of proline biosynthesis in Medicago truncatula reveals developmental and environmental specific features. Physiologia Plantarum 120: 442–450. 15032841 10.1111/j.0031-9317.2004.00251.x

[7] Deuschle K, Funck D, Forlani G, Stransky H, Biehl A, et al (2004) The role of Δ^1^-pyrroline-5-carboxylate dehydrogenase in proline degradation. The Plant Cell Online 16: 3413–3425. 10.1105/tpc.104.023622 PMC535882 15548746

[8] Kishor PBK, Hong Z, Miao GH, Hu CAA, Verma DPS (1995) Overexpression of delta-pyrroline-5-carboxylate synthetase increases proline production and confers osmotolerance in transgenic plants. Plant Physiology 108: 1387–1394. 12228549 10.1104/pp.108.4.1387 PMC157516

[9] Kumar V, Shriram V, Kavi Kishor PB, Jawali N, Shitole MG (2010) Enhanced proline accumulation and salt stress tolerance of transgenic indica rice by over-expressing *P5CSF129A* gene. Plant Biotechnology Reports 4: 37–48.

[10] Yamchi A, Jazii FR, Mousavi A, Karkhane AA (2007) Proline accumulation in transgenic tobacco as a result of expression of Arabidopsis Δ^1^-Pyrroline-5-carboxylate synthetase (P5CS) during osmotic stress. Journal of Plant Biochemistry and Biotechnology 16: 9–15.

[11] Vendruscolo ECG, Schuster I, Pileggi M, Scapim CA, Molinari HBC, et al (2007) Stress-induced synthesis of proline confers tolerance to water deficit in transgenic wheat. Journal of Plant Physiology 164: 1367–1376. 17604875 10.1016/j.jplph.2007.05.001

[12] Patterson JH, Newbigin E, Tester M, Bacic A, Roessner U (2009) Metabolic responses to salt stress of barley (*Hordeum vulgare* L.) cultivars, Sahara and Clipper, which differ in salinity tolerance. Journal of Experimental Botany 60: 4089–4103. 19666960 10.1093/jxb/erp243 PMC2755029

[13] Seki M, Umezawa T, Urano K, Shinozaki K (2007) Regulatory metabolic networks in drought stress responses. Current Opinion in Plant Biology 10: 296–302. 17468040 10.1016/j.pbi.2007.04.014

[14] Kiyosue T, Yoshiba Y, Yamaguchi-Shinozaki K, Shinozaki K (1996) A nuclear gene encoding mitochondrial proline dehydrogenase, an enzyme involved in proline metabolism, is upregulated by proline but downregulated by dehydration in Arabidopsis. The Plant Cell Online 8: 1323–1335. 10.1105/tpc.8.8.1323 PMC161248 8776899

[15] Huang Z, Long X, Kang J, Zhang Z, Zed R, et al (2012) Growth, photosynthesis and H^+^-ATPase activity in two Jerusalem artichoke varieties under NaCl-induced stress. Process Biochemistry 47: 591–596.

[16] Shannon MC, Grieve CM (1998) Tolerance of vegetable crops to salinity. Scientia Horticulturae 78: 5–38.

[17] Kosaric N, Cosentino GP, Wieczorek A, Duvnjak Z (1984) The Jerusalem artichoke as an agricultural crop. Biomass 5: 1–36.

[18] Hoagland DR, Arnon DI (1950) The water-culture method for growing plants without soil. Circular. California Agricultural Experiment Station 347.

[19] Bates LS, Waldren RP, Teare ID (1973) Rapid determination of free proline for water-stress studies. Plant and Soil 39: 205–207.

[20] Hayzer DJ, Leisinger TH (1980) The gene-enzyme relationships of proline biosynthesis in Escherichia coli. Journal of General Microbiology 118: 287–293. 6255065 10.1099/00221287-118-2-287

[21] Kim HR, Rho HW, Park JW, Park BH, Kim JS, et al (1994) Assay of ornithine aminotransferase with ninhydrin. Analytical Biochemistry 223: 205–207. 7887464 10.1006/abio.1994.1574

[22] Lutts S, Majerus V, Kinet JM (2002) NaCl effects on proline metabolism in rice (*Oryza sativa* L.) seedlings. Physiologia Plantarum 105: 450–458.

[23] Liang M, Hole D, Wu J, Blake T, Wu Y (2012) Expression and functional analysis of NUCLEAR FACTOR-Y, subunit B genes in barley. Planta: 1–13. 10.1007/s00425-011-1539-0 22042327

[24] Livak KJ, Schmittgen TD (2001) Analysis of relative gene expression data using Real-time quantitative PCR and the 2^−ΔΔCT^ method. methods 25: 402–408. 11846609 10.1006/meth.2001.1262

[25] Newton PJ, Myers BA, West DW (1991) Reduction in growth and yield of Jerusalem artichoke caused by soil salinity. Irrigation Science 12: 213–221.

[26] Da Rocha IMA, Vitorello VA, Silva JS, Ferreira-Silva SL, Viégas RA, et al (2012) Exogenous ornithine is an effective precursor and the δ-ornithine amino transferase pathway contributes to proline accumulation under high N recycling in salt-stressed cashew leaves. Journal of Plant Physiology 169: 41–49. 21903295 10.1016/j.jplph.2011.08.001

[27] Kishor PBK, Sangam S, Amrutha RN, Laxmi PS, Naidu KR, et al (2005) Regulation of proline biosynthesis, degradation, uptake and transport in higher plants: its implications in plant growth and abiotic stress tolerance. Current Science 88: 424–438.

[28] Walton EF, Podivinsky E, Wu RM, Reynolds PHS, Young LW (1998) Regulation of proline biosynthesis in kiwifruit buds with and without hydrogen cyanamide treatment. Physiologia Plantarum 102: 171–178.

[29] Roosens NHCJ, Thu TT, Iskandar HM, Jacobs M (1998) Isolation of the ornithine-δ-aminotransferase cDNA and effect of salt stress on its expression in *Arabidopsis thaliana* . Plant Physiology 117: 263. 9576796 10.1104/pp.117.1.263 PMC35011

[30] Kimura T, Nakano T, Taki N, Ishikawa M, Asami T, et al (2001) Cytokinin-induced gene expression in cultured green cells of Nicotiana tabacum identified by fluorescent differential display. Bioscience, Biotechnology, and Biochemistry 65: 1275–1283. 10.1271/bbb.65.1275 11471724

[31] Su M, Li XF, Ma XY, Peng XJ, Zhao AG, et al (2011) Cloning two *P5CS* genes from bioenergy sorghum and their expression profiles under abiotic stresses and MeJA treatment. Plant Science 181: 652–659. 21958707 10.1016/j.plantsci.2011.03.002

[32] Ribarits A, Abdullaev A, Tashpulatov A, Richter A, Heberle-Bors E, et al (2007) Two tobacco proline dehydrogenases are differentially regulated and play a role in early plant development. Planta 225: 1313–1324. 17106685 10.1007/s00425-006-0429-3

[33] Saadia M, Jamil A, Akram NA, Ashraf M (2012) A study of proline metabolism in Canola (*Brassica napus* L.) seedlings under salt stress. Molecules 17: 5803–5815. 22592086 10.3390/molecules17055803 PMC6268620

[34] Misra N, Gupta AK (2005) Effect of salt stress on proline metabolism in two high yielding genotypes of green gram. Plant Science 169: 331–339.

[35] Surabhi GK, Reddy AM, Kumari GJ, Sudhakar C (2008) Modulations in key enzymes of nitrogen metabolism in two high yielding genotypes of mulberry (*Morus alba* L.) with differential sensitivity to salt stress. Environmental and Experimental Botany 64: 171–179.

[36] Xue X, Liu A, Hua X (2009) Proline accumulation and transcriptional regulation of proline biothesynthesis and degradation in *Brassica napus* . BMB Reports 42: 28–34. 19192390 10.5483/bmbrep.2009.42.1.028

[37] Shahbaz M, Ashraf M, Akram NA, Hanif A, Hameed S, et al (2011) Salt-induced modulation in growth, photosynthetic capacity, proline content and ion accumulation in sunflower (*Helianthus annuus* L.). Acta Physiologiae Plantarum 33: 1113–1122.

[38] Silva-Ortega CO, Ochoa-Alfaro AE, Reyes-Agüero JA, Aguado-Santacruz GA, Jiménez-Bremont JF (2008) Salt stress increases the expression of *P5CS* gene and induces proline accumulation in cactus pear. Plant Physiology and Biochemistry 46: 82–92. 18054243 10.1016/j.plaphy.2007.10.011

[39] Dobrá J, Vanková R, Havlová M, Burman AJ, Libus J, et al (2011) Tobacco leaves and roots differ in the expression of proline metabolism-related genes in the course of drought stress and subsequent recovery. Journal of Plant Physiology 168: 1588–1597. 21481968 10.1016/j.jplph.2011.02.009

[40] Wang K, Liu Y, Dong K, Dong J, Kang J, et al (2011) The effect of NaCl on proline metabolism in Saussurea amara seedlings. African Journal Biotechnology 10: 2886–2893.

[41] Delauney AJ, Verma DPS (2002) Proline biosynthesis and osmoregulation in plants. The Plant Journal 4: 215–223.

[42] Roy D, Bhunia A, Basu N, Banerjee SK (1992) Effect of NaCl-salinity on metabolism of proline in salt-sensitive and salt-resistant cultivars of rice. Biologia Plantarum 34: 159–162.

[43] Lutts S, Majerus V, Kinet JM (2002) NaCl effects on proline metabolism in rice (*Oryza sativa* L.) seedlings. Physiologia Plantarum 105: 450–458.

[44] Székely G, Ábrahám E, Cséplő Á, Rigó G, Zsigmond L, et al (2007) Duplicated *P5CS* genes of Arabidopsis play distinct roles in stress regulation and developmental control of proline biosynthesis. The Plant Journal 53: 11–28. 17971042 10.1111/j.1365-313X.2007.03318.x

[45] Funck D, Stadelhofer B, Koch W (2008) Ornithine-δ-aminotransferase is essential for arginine catabolism but not for proline biosynthesis. BMC Plant Biology 8: 40. 18419821 10.1186/1471-2229-8-40 PMC2377265

[46] You J, Hu H, Xiong L (2012) An ornithine δ-aminotransferase gene *OsOAT* confers drought and oxidative stress tolerance in rice. Plant Science 197: 59–69. 23116672 10.1016/j.plantsci.2012.09.002

[47] Miller G, Stein H, Honig A, Kapulnik Y, Zilberstein A (2005) Responsive modes of Medicago sativa proline dehydrogenase genes during salt stress and recovery dictate free proline accumulation. Planta 222: 70–79. 15809861 10.1007/s00425-005-1518-4

[48] Funck D, Eckard S, Müller G (2010) Non-redundant functions of two proline dehydrogenase isoforms in Arabidopsis. BMC Plant Biology 10: 70. 20403182 10.1186/1471-2229-10-70 PMC3095344

