# Supplementary figures and images for: Salt Stress Encourages Proline Accumulation by Regulating Proline Biosynthesis and Degradation in Jerusalem Artichoke Plantlets
Source: PLoS One. 2013 Apr 29;8(4):e62085. doi: 10.1371/journal.pone.0062085 (PMC3639250; doi:10.1371/journal.pone.0062085)

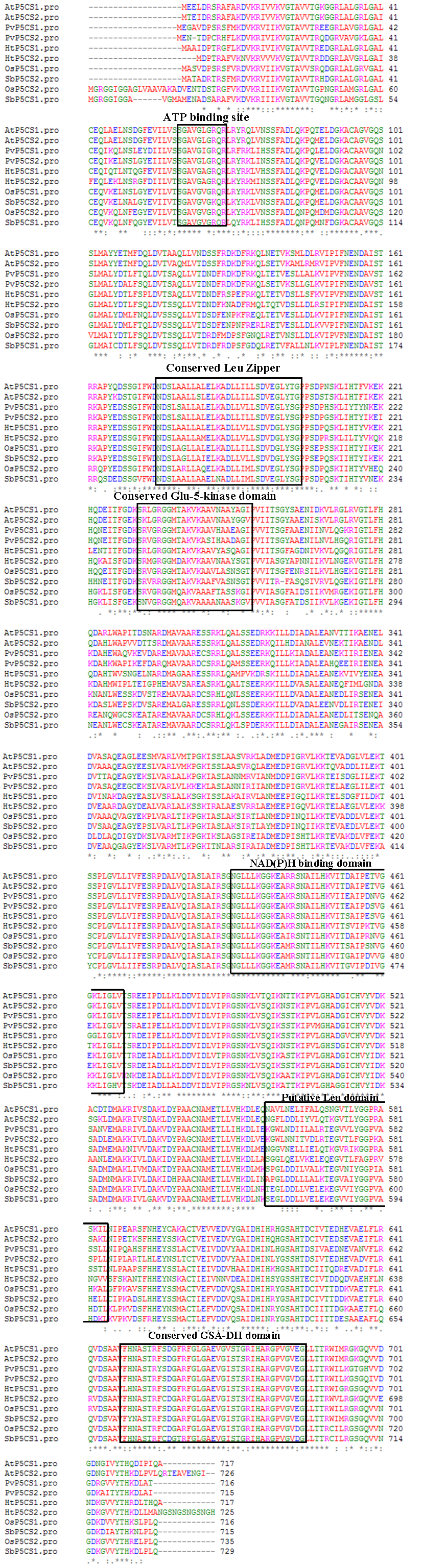

Supplement: Figure S1 — Amino acid sequence alignments of HtP5CS1 and HtP5CS2 . Alignment was performed using the ClustalW program. The following sequences with corresponding accession number were used for bioinformatic analysis: AtP5CS1 (NM_129539), AtP5CS2 (NM_115419.4); OsP5CS1 (D49714.1), OsP5CS2 (NM_001051337); PvP5CS1 (EU340347), PvP5CS2 (EU407263); SbP5CS1 (GQ377719), SbP5CS2 (GQ377720). Asterisk, semicolon and dot represent the amino that are “identical”, “conserved substitution” and “semi-conserved substitution”, respectively. Boxed sequences showed conserved putative ATP and NAD(P)H-binding sites, GK and GSA-DH domains, and putative Leu-rich regions. (TIF) [file pone.0062085.s001.tif]

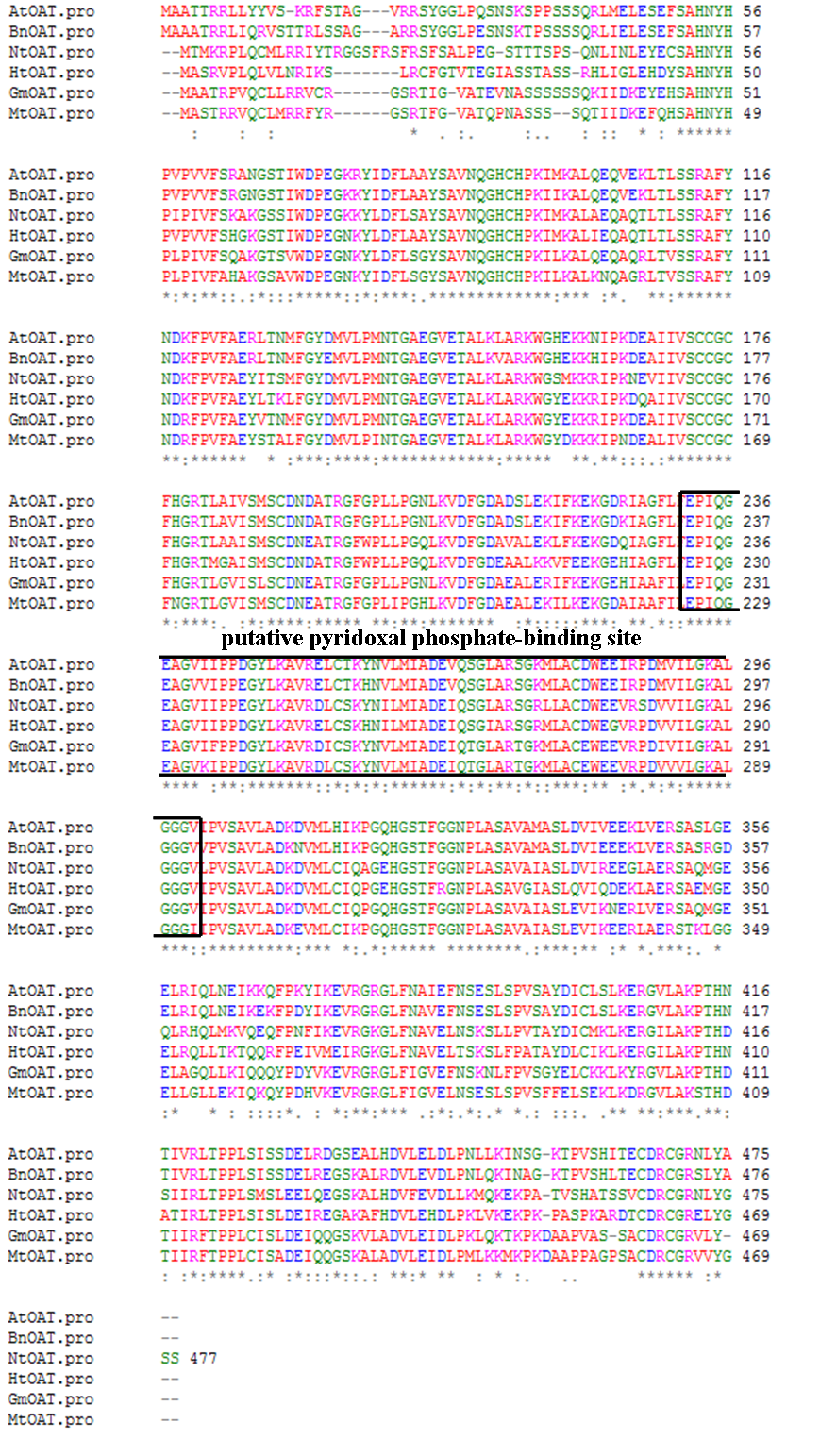

Supplement: Figure S2 — Amino acid sequence alignments of HtOAT . Alignment was performed using the ClustalW program. The following sequences with corresponding accession number were used for bioinformatic analysis: AtOAT (NM_123987.3); BnOAT (EU375566.1); GmOAT (NM_001250221.1); MtOAT (AJ278819); NtOAT (ADM47437). Asterisk, semicolon and dot represent the amino that are “identical”, “conserved substitution” and “semi-conserved substitution”, respectively. Boxed sequences showed putative pyridoxal phosphate-binding domain. (TIF) [file pone.0062085.s002.tif]

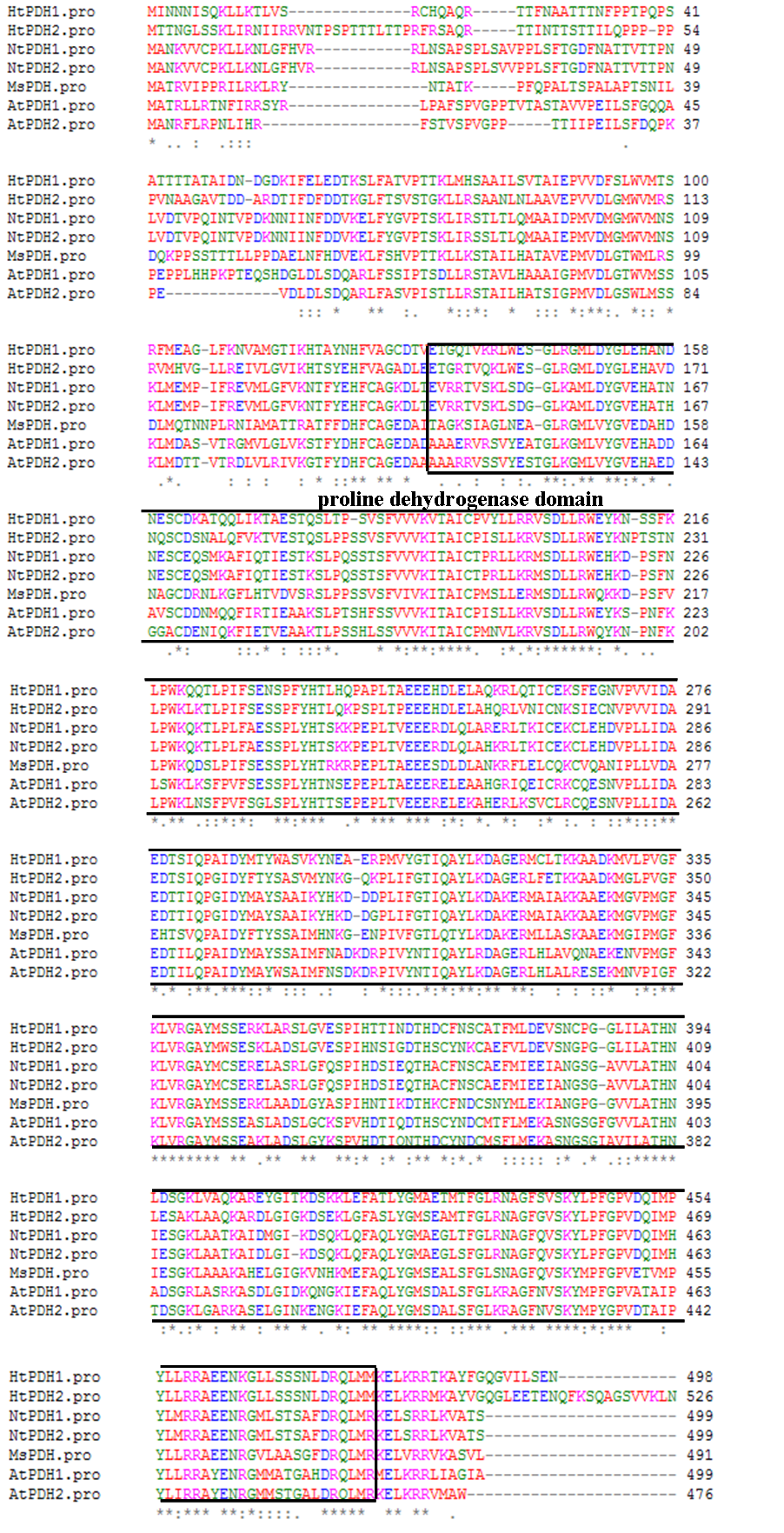

Supplement: Figure S3 — Amino acid sequence alignments of HtPDH1 and HtPDH2 . Alignment was performed using the ClustalW program. The following sequences with corresponding accession number were used for bioinformatic analysis: AtPDH1 (NM_113981.5), AtPDH2 (NM_123232.2); MsPDH (AY556386.1); NtPDH1 (AY639145.1), NtPDH2 (AY639146.1). Asterisk, semicolon and dot represent the amino that are “identical”, “conserved substitution” and “semi-conserved substitution”, respectively. Boxed sequences showed proline dehydrogenase domain. (TIF) [file pone.0062085.s003.tif]

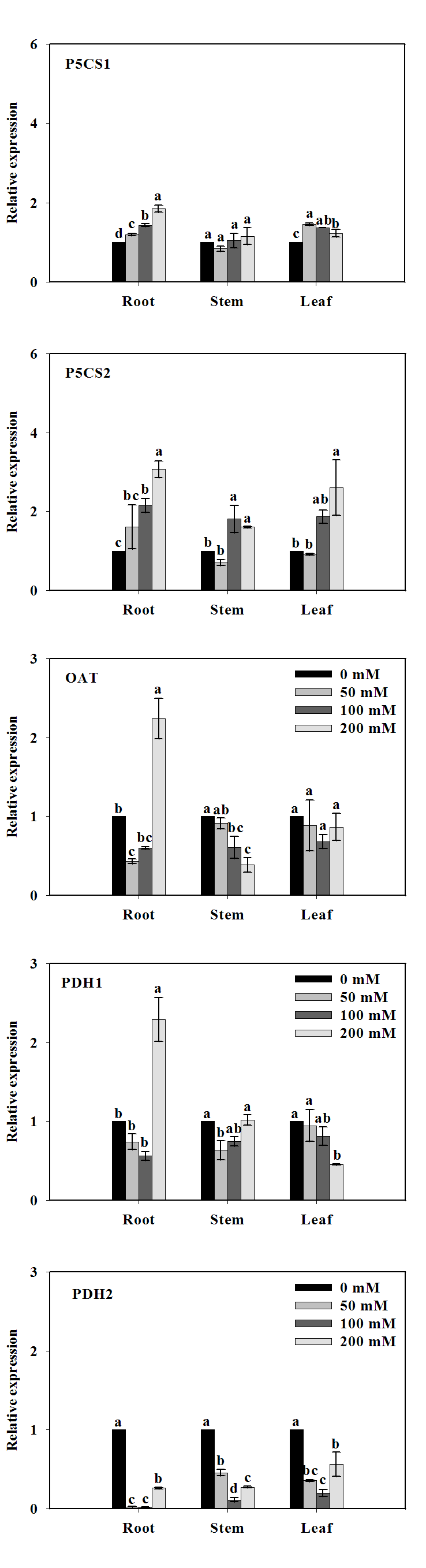

Supplement: Figure S4 — Expression profiles of HtP5CS1, HtP5CS2, HtOAT, HtPDH1, and HtPDH2 from roots, stems, and leaves under different NaCl dosage stresses at 12 h. 30-day-old Jerusalem artichoke plantlets were treated with 0, 50, 100 and 200 mM NaCl (see Materials and Methods) for 12 h, respectively. All treatments had three biological replicates. Total RNA was extracted from roots, stems and leaves for quantitative PCR (qPCR) analysis. Transcript levels were first normalized to the level of a control gene actin (HtActin). The normalized transcript levels were then compared between treatments (50, 100 and 200 mM NaCl) and control (0 mM NaCl) to obtain a fold change. Values represent means ± SE of three independent experiments. Significant differences (P≤0.05) between treatments are indicated by different letters. Values represent means ± SE of three independent experiments. Significant differences (P≤0.05) between treatments are indicated by different letters. (TIF) [file pone.0062085.s004.tif]
