# Supplementary material for: Salt Stress Encourages Proline Accumulation by Regulating Proline Biosynthesis and Degradation in Jerusalem Artichoke Plantlets
Source: PLoS One. 2013 Apr 29;8(4):e62085. doi: 10.1371/journal.pone.0062085 (PMC3639250; doi:10.1371/journal.pone.0062085)
Supplement: Table S1 — Primers for gene cloning of HtP5CS1, HtP5CS2, HtOAT, HtPDH1, and HtPDH2 . (DOC) [file pone.0062085.s005.doc]

**Supplement Table 1** Primers for gene cloning of *Ht P5CS1, Ht P5CS2, Ht OAT, Ht PDH1, and Ht PDH2*

| **Gene** | **Primer number** | **Primer(5’-3’)** |
| --- | --- | --- |
| *Ht P5CS1* | 1 | F: GCAGATACTCAAACCCTA |
|  |  | R: TTCTTCCCTCTCCAACAA |
| *Ht P5CS2* | 2 | F: GTCAAGCGTGTAGTCGTC |
|  |  | R: GGTGTAAACAACTCCCTT |
|  | 3 | GSP: TGATGGGGCTCGGTTTGGACTCGGA |
|  |  | NGSP: CGAGGTCCAGTTGGCGTAGAGGG |
|  | 4 | F: CAAAAAACCTCACATTCGA |
|  |  | R: CTTACTGTGTTCGTTTAATAT |
| *Ht OAT* | 5 | F: CACTCTGGGACGATTCAACA |
|  |  | R：AATAACCGCCTCAACTCCTC |
|  | 6 | F: GGATAGAGTGTTTCGTAA |
|  |  | R: AATAACCTAGCATTGAGA |
| *Ht PDH1* | 7 | F: GCCTTAAACTTTCACCCG |
|  |  | R: TGTGACTTGTGACAGCCA |
| *Ht PDH2* | 8 | F: TTATTCAGCCGAAAAACTT |
|  |  | R: ATTATTCCAAAATCCCCAT |
