# Supplementary material for: Salt Stress Encourages Proline Accumulation by Regulating Proline Biosynthesis and Degradation in Jerusalem Artichoke Plantlets
Source: PLoS One. 2013 Apr 29;8(4):e62085. doi: 10.1371/journal.pone.0062085 (PMC3639250; doi:10.1371/journal.pone.0062085)
Supplement: Table S2 — Primers for qPCR of HtP5CS1, HtP5CS2, HtOAT, HtPDH1, and HtPDH2 . (DOC) [file pone.0062085.s006.doc]

**Supplement Table 2** Primers for qPCR of *Ht P5CS1, Ht P5CS2, Ht OAT, Ht PDH1, and Ht PDH2*

| **Gene** | **Primer (5’-3’)** |
| --- | --- |
| *Ht P5CS1* | F: CTGGAAGCAAATGAAAAAG |
|  | R: TGAGAGCCAAACGAGAGACTAA |
| *Ht P5CS2* | F: GGCAATGGAACTGAAGGC |
|  | R: CCTCTTCCCATCCTTGACTTA |
| *Ht OAT* | F: TTGAGCAGGCACAAACCC |
|  | R: TCATTGGCAGAACCATATCG |
| *Ht PDH1* | F: ATGCTGGATTATGGGTTGG |
|  | R: CAGAAGGAGTAAGAGATTGGGTG |
| *Ht PDH2* | F: GGGTTTGCTTCACTTTATGGT |
|  | R: TTATTCTCTTCGGCTCGTCTC |
| *Ht Actin* | F: ATGTATGTAGCCATCCAGG |
|  | R: TGTTAGGTCACGCCCAG |
